# Coloring-Based Serious Game in Chinese College Students With Alexithymia: Prospective Longitudinal Exploratory Mixed Methods Study

**DOI:** 10.2196/83540

**Published:** 2026-08-19

**Authors:** Shuzhan Liu, Haoyong Deng, Jing Chen, Qi Wang

**Affiliations:** 1 College of Design and Innovation, Tongji University, 1239 Siping Road, Shanghai, 200092, China, 86 18321024151; 2 School of Nursing and Health Management, Shanghai University of Medicine and Health Sciences, Shanghai, China

**Keywords:** alexithymia, serious game, digital art therapy, emotion regulation, mixed methods, Chinese college students

## Abstract

**Background:**

Alexithymia, characterized by difficulty identifying, describing, and expressing emotions, is prevalent among Chinese college students. Traditional art therapies face barriers, such as high psychological thresholds and limited accessibility. Accessible, low-threshold digital tools are needed to help address this challenge. Coloring the Emoji, a coloring-based serious game designed as a digital art therapy, offers a low-threshold tool to support emotion regulation for this population.

**Objective:**

This study aimed to evaluate the effectiveness of Coloring the Emoji as a digital art therapy in improving alexithymia symptoms and examine the user experience of the game, thereby providing empirical support for the application of coloring-based digital art therapy in alexithymia intervention.

**Methods:**

A prospective, exploratory convergent parallel mixed methods longitudinal design was used, involving a 15-day daily game intervention with Chinese college students exhibiting alexithymic tendencies. Participants were purposively sampled from 332 prescreened students. Eligibility criteria included age 18‐30 years, basic smartphone operation, and no ongoing psychotherapy or psychiatric medication. In total, 20 participants completed the intervention. Quantitative assessments used the 20-item Toronto Alexithymia Scale to measure alexithymia and the Game Experience Questionnaire to assess user experience. Statistical analyses comprised Wilcoxon signed-rank tests for pre-post comparisons, Mann-Whitney *U* tests for sex, education, and major comparisons, and Kruskal-Wallis H tests for 20-item Toronto Alexithymia Scale pretest severity comparisons. The qualitative component consisted of 3 longitudinal semistructured interviews conducted across the intervention period (days 1, 8, and 15), analyzed using Colaizzi’s phenomenological method and thematic analysis to explore emotion regulation dynamics, user experience, and in-depth mechanisms underlying intervention changes.

**Results:**

Total TAS-20 scores decreased significantly post intervention (*Z*=2.425; *P*=.02), with a median reduction of 3.00 points (95% CI 1.00‐4.50, 5000 bootstrap replications). The Difficulty Identifying Feelings subscale showed a marginal improvement (*Z*=1.826; *P*=.07). Males exhibited significantly greater Difficulty Identifying Feelings reduction (*Z*=−2.137; *P*=.03), and participants with severe baseline alexithymia showed greater improvement in Externally Oriented Thinking (H=8.233; *P*=.02). Game Experience Questionnaire scores indicated high competence and sensory and imaginative immersion. Qualitative results confirmed 2 major themes and 10 subthemes; visual coloring practice helped participants externalize vague emotions, differentiated engagement habits explained subgroup discrepancies, and low-pressure interaction supported sustainable emotional regulation. This study further integrated quantitative and qualitative outcomes through subgroup comparative analysis, revealing distinct intervention response patterns across sex and alexithymia severity.

**Conclusions:**

Coloring the Emoji is an effective, acceptable intervention for improving emotional identification in Chinese college students with alexithymia. Its innovation lies in a localized low-threshold design that meets daily emotional needs. Unlike mainstream Western interventions emphasizing open emotional expression and social sharing, this tool adapts to the reserved emotional disclosure norms of Chinese college students. It advances the field by supplementing culturally contextualized evidence for nonverbal digital art therapy. As a low-stress, demedicalized solution, it provides a scalable, feasible supplement to traditional mental health services for campus emotional regulation.

## Introduction

Alexithymia, a concept introduced by psychiatrist Peter Sifneos in 1973 [1], refers to significant difficulties in identifying, describing, and expressing emotions, often accompanied by reduced imaginative capacity and limited symbolic thinking [2]. Alexithymia has broad implications for both psychological and physiological health [3,4]. On one hand, it is closely associated with various psychosomatic conditions, such as hypertension, asthma, and ulcerative colitis [5-7]. On the other hand, alexithymia undermines psychotherapy by weakening the therapeutic alliance, leading to higher dropout rates and reduced responsiveness to approaches that rely on emotional insight, such as psychodynamic therapy [8-10]. Recent studies further indicate that alexithymia is increasingly prevalent among young adults, particularly in Chinese college students, and is closely associated with elevated anxiety, depression, and academic burnout [11-13]. Our previous survey of Chinese college students (n=332) highlights the severity of the issue: 12.05% (40/332) of respondents exhibited clinically significant alexithymia (20-item Toronto Alexithymia Scale [TAS-20]≥70) [14-16], with a mean score of 59.90 (SD 8.15), approaching the clinical threshold (TAS-20≥61). Existing studies have primarily focused on the epidemiological characteristics and negative outcomes of alexithymia, but few have developed localized, low-threshold, and easily accessible intervention programs suitable for Chinese college students [17,18]. These findings highlight the severity of alexithymia in this population and the need for targeted attention.

Coloring-based art therapy offers a simple and structured activity that allows college students to associate emotions with colors and express them in a low-pressure setting [19]. Studies have suggested that coloring tasks can reduce anxiety, promote relaxation, and offer a manageable pathway for emotional expression [20], which may be especially beneficial for college students who struggle with verbal articulation [21,22]. Emerging evidence has also highlighted coloring as a low-threshold, nonverbal approach suitable for individuals with limited emotional insight [23-25]. However, the broader applicability of coloring-based art therapy is limited by attitudinal barriers such as shame and embarrassment [26], reduced initiative due to emotional avoidance [27], and practical obstacles including time, spatial, and financial constraints [28]. These limitations underscore the need for delivery formats that increase feasibility and maintain engagement [29,30].

In this context, digital adaptations of coloring-based art therapy have emerged as a promising alternative, integrating psychological principles with interactive design to create accessible platforms for emotion regulation [31,32]. Digital coloring games offer an intuitive medium that combines structured tasks with interactive feedback, promoting consistent participation and overcoming barriers associated with traditional approaches. Through real-time feedback and immersive experiences, these games encourage users to engage more deeply with their emotions [33,34]. Compared with traditional methods, coloring games also offer greater enjoyment and accessibility, can enhance user initiative, maintain long-term engagement, and support the transition from unconscious to conscious emotional expression [35,36]. Notably, few digital interventions have been systematically developed and empirically tested for alexithymia in Chinese college students, leaving a key gap in evidence-based digital mental health tools [37].

Based on these considerations, we developed Coloring the Emoji, a serious game for emotional recognition and expression. The game was built upon our previous intervention framework derived from expert interviews and codesign, which emphasized visual expression, daily integration, implicit emotional expression, and psychological safety [38]. Drawing on expert interviews and a codesign process, the game incorporates the principles of color psychology [39], dimensional emotion models [40,41], and symbolic association [42,43] into a safe and intuitive interactive process, thereby addressing the core difficulties of alexithymia in identifying, associating, and expressing emotions [44,45].

Therefore, this study aimed to empirically validate the game through a prospective, exploratory mixed methods longitudinal design, with the following objectives:

1. To evaluate the effectiveness of Coloring the Emoji in reducing alexithymia symptoms among Chinese college students
2. To examine the user experience of the game as a daily emotion regulation tool
3. To provide empirical support for the application of coloring-based digital art therapy in alexithymia intervention

The findings are expected to inform the practical application of such tools and provide an empirical basis for the improvement and wider application of coloring-based digital art therapy in the future.

## Methods

### Research Design Overview

This study used a prospective, exploratory convergent parallel mixed methods longitudinal design to evaluate the effectiveness and user experience of Coloring the Emoji, a coloring-based serious game developed as a digital art therapy for alexithymia [46]. This design integrated quantitative outcomes and qualitative data from the same sample to provide a comprehensive, triangulated understanding of the game’s effectiveness and user experience. Participants were recruited based on our preliminary survey of 332 Chinese college students. From this pool, students with elevated alexithymia scores and a high willingness to participate were invited to a 15-day intervention. This duration was selected based on previous research demonstrating that smartphone-based psychological interventions can yield observable changes within a 2-week window [47]. Quantitative assessment involved pre- and posttest comparisons of the TAS-20 [48] and the posttest Game Experience Questionnaire (GEQ) [49]. In parallel, qualitative data were collected through semistructured interviews on days 1, 7, and 15 to gain in-depth insights into participants’ emotional engagement and their evolving perceptions of the game.

### Participant Recruitment

Participant recruitment began in October 2024 via online and offline channels, with peer referrals encouraged. To minimize stigma and expectancy bias, recruitment materials framed the project as a digital game study without mentioning mental health-related terminology. This study used purposive sampling [50]. Eligibility criteria for participants included an age range of 18‐30 years, a TAS-20 score of 52 or higher, and basic smartphone operation ability. Those receiving ongoing psychotherapy, taking psychiatric medication, or unable to finish intervention tasks were excluded. Finally, a total of 20 participants were enrolled in the 15-day game-based intervention experiment (Table 1). The same sample was used for both quantitative and qualitative analyses.

**Table 1.** Demographic characteristics and behavior data of study participants.^a,b^

Characteristics	Statistics, n (%)
Sex	
Male	9 (45)
Female	11 (55)
Education level	
Undergraduate	8 (40)
Postgraduate	12 (60)
TAS-20^a^ pretest severity level	
Borderline alexithymia (52-60)	5 (25)
Definite alexithymia (61-69)	12 (60)
Severe alexithymia (≥70)	3 (15)
Academic major	
Design	13 (65)
Nondesign^b^	7 (35)

a TAS-20: 20-item Toronto Alexithymia Scale.

b Non-design majors: Psychology, Business, Computer Science, and Engineering.

### Data Collection

#### Study Tool

**Figure 1.**
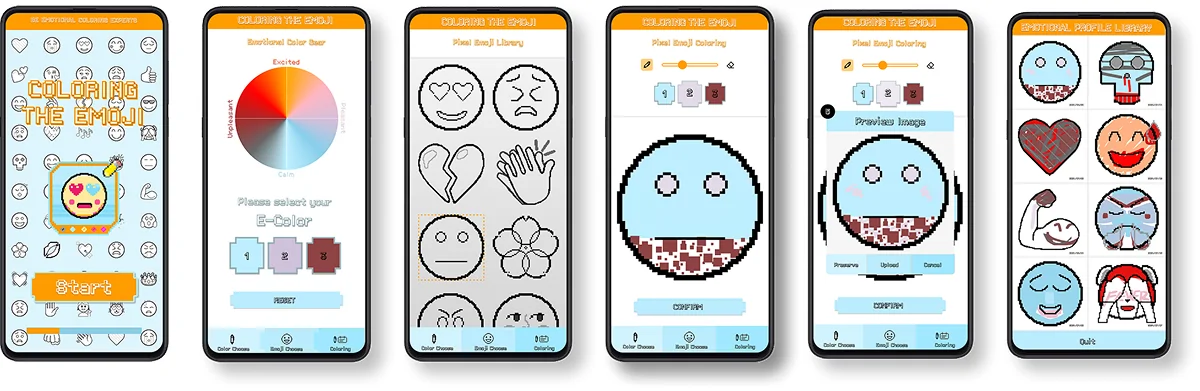
Interface and core functions of Coloring the Emoji - a coloring-based serious game for emotion regulation.

The primary instrument in this study was Coloring the Emoji, a coloring-based serious game developed to support Chinese college students with alexithymia in recognizing, externalizing, and expressing their emotions through intuitive coloring tasks (Figure 1). Compatible with iOS (Apple Inc) and Android (Google) mobile platforms, the game includes 3 main interactive stages—color selection, pattern selection, and free coloring—aiming to create a nonverbal, low-pressure emotional exploration environment. The overall interface uses a clean visual design and pixel art aesthetic to enhance ease of use and minimize defensive reactions.

In addition to the game itself, supplementary materials were prepared to facilitate participant onboarding and minimize technical barriers. These included an introduction video and a user manual (Multimedia Appendix 1). The video provided background on the game’s development, theoretical basis, and intended therapeutic goals, while the manual offered practical guidance on installation, interface navigation, and key features [51]. All participants received installation instructions in advance and were guided to install the game on their personal devices. They were also provided with an introductory video and a user manual to ensure a clear understanding of the game before starting the experiment.

#### Study Procedures

After providing informed consent, participants were guided step by step through the study protocol. On the first day of the experiment (December 8, 2024), participants received instructions for installing and using Coloring the Emoji via an introduction video and user manual. They also completed a pretest assessment using the TAS-20 [48] and a semistructured interview (Multimedia Appendix 2) via Zoom (Zoom Communications Inc) regarding their use of emotion regulation tools. After completing the game setup, all participants began a 15-day intervention, during which they were instructed to engage with the game daily and use its core functions to record and reflect on their emotions.

During the experiment, participants participated in 2 subsequent semistructured interviews via Zoom on day 8 and day 15 to provide feedback on their experiences and perceptions of the game. Each interview was conducted by one of the researchers, with a separate team member taking detailed notes. All interviews were audio-recorded, transcribed verbatim, and checked for accuracy before being anonymized. At the end of the experiment (December 23, 2024), participants completed posttest assessments, including the TAS-20 [48] and the GEQ [49]. All procedures were standardized across participants, and both qualitative and quantitative datasets were anonymized, securely stored, and archived for subsequent reporting.

#### Quantitative Data Collection

The primary effectiveness measure was change in alexithymia scores, assessed pretest and posttest using the TAS-20 [48]. This scale comprises 3 subscales: Difficulty Identifying Emotions (DIF), Difficulty Describing Emotions, and Externally Oriented Thinking (EOT). Items are rated on a 5-point Likert scale, ranging from 1 (strongly disagree) to 5 (strongly agree). A total score of 52 or above is considered clinically significant: scores between 52 and 60 represent borderline alexithymia, scores of 61 or above indicate definite alexithymia, and individuals with scores of 70 or above may also experience co-occurring complications.

User experience was assessed at posttest using the 33-item GEQ [49] to assess user experience and provide a multidimensional quantitative overview of participants’ psychological engagement and emotional state during gameplay. This questionnaire is a validated instrument for digital games that covers 7 core dimensions: competence, sensory and imaginative immersion, flow, tension or annoyance, challenge, negative affect, and positive affect. Items are rated on a 5-point Likert scale ranging from 0 (not at all) to 4 (extremely). The score for each component is calculated as the average of its constituent items, with higher scores indicating a stronger experience of that specific dimension.

#### Qualitative Data Collection

The same participants completed 3 semistructured interviews on day 1, day 8, and day 15 via Zoom. Repeated interviews across different intervention stages were designed to capture dynamic, longitudinal changes in participants’ emotional perceptions, game engagement, and emotional regulation experiences, rather than relying solely on 1-time retrospective feedback. The semistructured interview guide was formally developed based on the research objectives, relevant literature on art therapy, serious game intervention, and alexithymia intervention. The initial draft was reviewed, revised, and finalized by the research team to ensure the relevance of the interview content. Interviews explored emotional experiences, perceived changes in emotion regulation, engagement with the game, and user experience. Interviews were audio-recorded, transcribed verbatim, cross-checked for accuracy, and anonymized before data analysis.

### Statistical Analysis

#### Quantitative Analysis

All quantitative data were analyzed using SPSS Statistics (version 27; IBM Corp). Descriptive statistics were first computed, with demographic variables reported as frequencies (n, %), gaming behavior data measures as means and SDs, and questionnaire scores as medians and IQRs. Since some of the data exhibited a nonnormal distribution, this study used nonparametric tests. To evaluate the intervention effect, the Wilcoxon signed-rank test was used to compare the pre- and postintervention differences in participants’ TAS-20 scores. For user experience evaluation, descriptive analysis was conducted on the GEQ outcomes. Furthermore, to explore the influence of demographic variables on the experimental results, the Mann-Whitney *U* test was used to compare the effects of sex, education level, and major on the difference in TAS-20 scores (pre- and postintervention difference) and each dimension of the GEQ. For variables with more than 2 groups, such as the initial alexithymia severity (TAS-20 pretest level), the Kruskal-Wallis H test was applied to identify possible differences in TAS-20 score changes and GEQ dimensions across groups.

#### Qualitative Analysis

Qualitative data were analyzed using NVivo 15 (Lumivero) following Colaizzi’s [52] 7-step phenomenological method to ensure a systematic and rigorous interpretation. Each interview was transcribed and cross-checked by 2 researchers (SL and HD) for accuracy. To establish methodological rigor and trustworthiness, the primary researcher and an additional researcher, both trained in qualitative theory, independently conducted the analysis through a structured process: (1) familiarizing with the data through repeated reading, (2) extracting significant statements, (3) formulating meanings by condensing codes while retaining original intent, (4) categorizing codes into clusters, (5) developing subthemes and themes, (6) formulating the fundamental structure of the phenomenon, and (7) validating findings through participant member-checking. To ensure high intercoder reliability and minimize personal bias, the 2 coders (SL and HD) cross-verified their results independently, with a third senior researcher (JC) reviewing the entire process to resolve any discrepancies.

#### Mixed Methods Integration

Quantitative and qualitative findings were integrated via triangulation using a subgroup-comparative joint display approach. Quantitative results (TAS-20 and GEQ) were used to identify statistically significant changes, while qualitative themes were used to explain the underlying reasons, mechanisms, and user experiences behind these changes. The 2 datasets were combined to provide a holistic interpretation of intervention effectiveness, particularly to clarify how and why outcomes varied across different participant subgroups.

### Validity, Reliability, and Methodological Integrity

Quantitative rigor was ensured through the use of validated scales (TAS-20 and GEQ) and standard nonparametric statistical procedures. Qualitative rigor was established via independent double coding, member checking, peer debriefing, and a clear audit trail. Mixed methods rigor was achieved through concurrent data collection, consistent sampling, and systematic triangulation of quantitative and qualitative findings.

### Ethical Considerations

This study was reviewed and approved by the Institutional Review Board of Tongji University (approval TJDXSR076). Before study initiation, each participant received an institutionally approved electronic informed consent form that described the study’s purpose, procedures, tasks, potential risks, and anticipated benefits. Written consent was obtained electronically to confirm voluntary participation. Participants were informed of their right to withdraw at any stage without penalty. After the intervention, all participants received customized gifts based on their coloring creations during the game as compensation for participation, with each gift valued at CNY 30 (approximately US $4.15). To safeguard privacy, all data were anonymized and deidentified before analysis, and personal information was securely stored and accessible only to the research team. Confidentiality was strictly maintained throughout the study, and all results are reported at the group level to prevent individual identification. No individual participants are identifiable in any figures, images, or supplementary materials; all visual and textual data have been fully anonymized.

## Results

### Recruitment Outcomes

**Figure 2.**
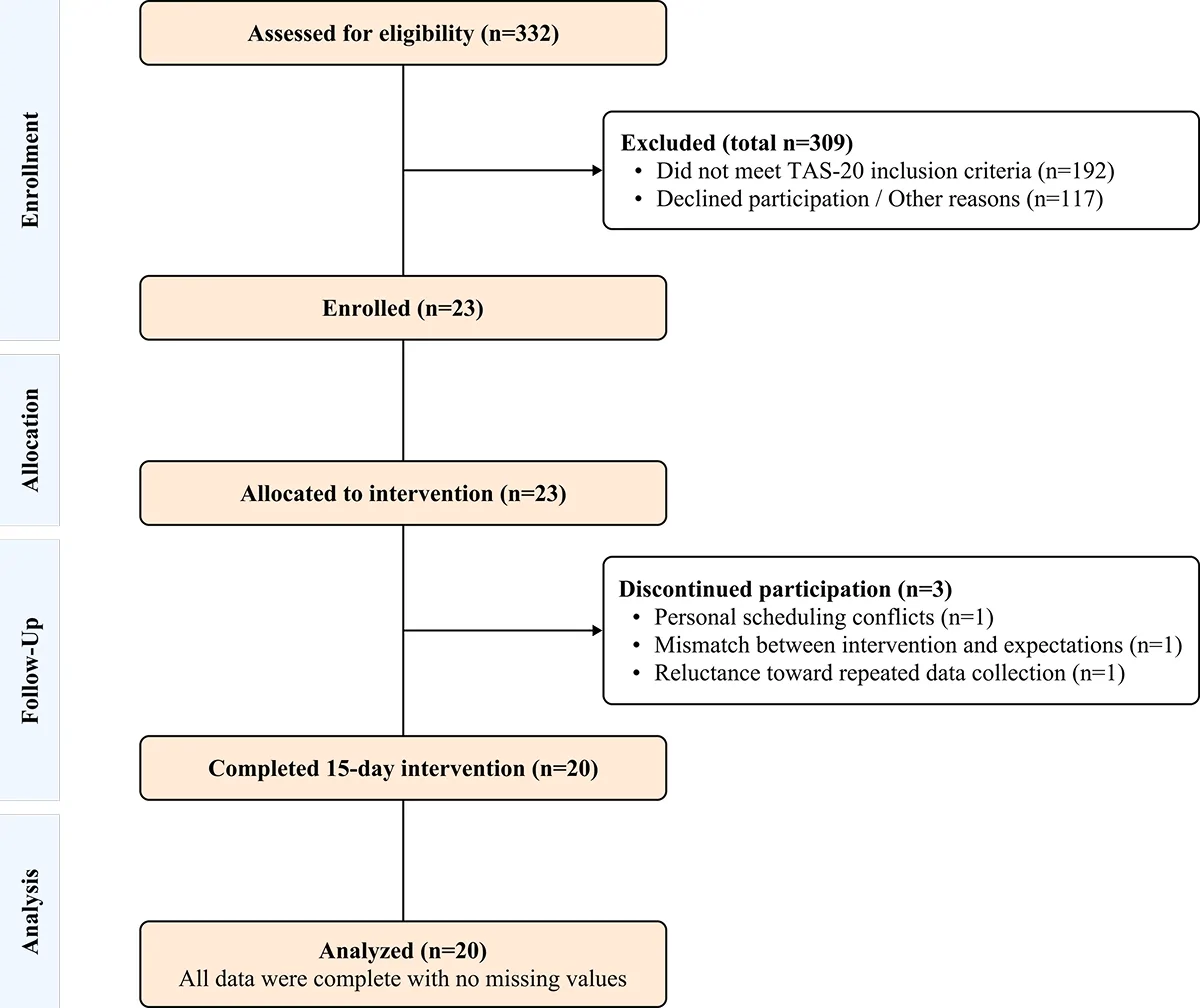
Participant flowchart of a game intervention study for Chinese college students with alexithymia. TAS-20: 20-item Toronto Alexithymia Scale.

For participant recruitment and screening, a total of 332 college students were initially assessed for eligibility. Of these, 309 individuals were excluded due to unqualified TAS-20 scores, refusal to participate, or other ineligible conditions. Initially, 23 participants were enrolled in the 15-day game-based intervention; however, 3 withdrew during the study due to scheduling conflicts, mismatched expectations about the intervention, or resistance to repeated data collection. Ultimately, 20 participants completed the full intervention with complete datasets, which were used for the final analysis (Figure 2). Given its exploratory pilot design, no a priori power analysis was conducted. This study aimed to collect preliminary data and inform sample planning for future larger trials.

### Quantitative Results

#### Results of TAS-20 and GEQ

**Figure 3.**
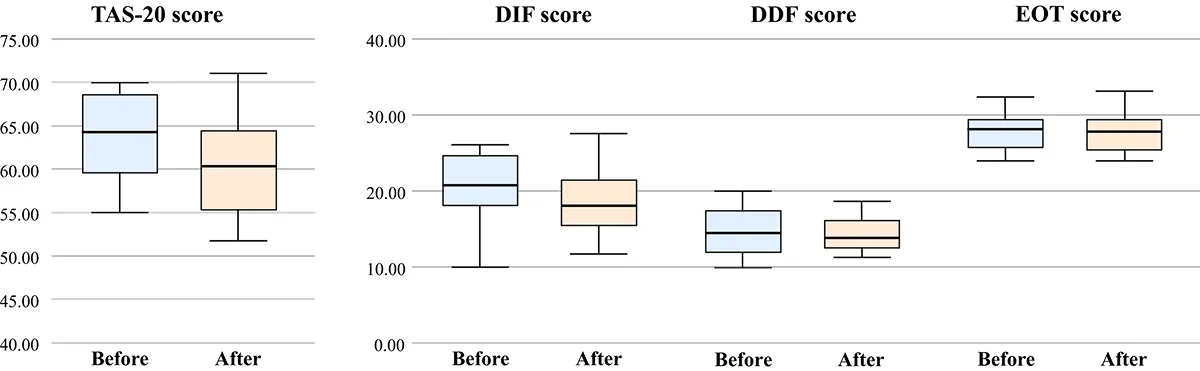
Boxplots of TAS-20 total and subscale scores pre- and post-intervention. DDF: Difficulty Describing Feelings; Difficulty Identifying Feelings; Externally Oriented Thinking; TAS-20: 20-item Toronto Alexithymia Scale.

The TAS-20 total score showed a significant reduction from pretest (median 64.00, IQR 59.50‐68.00) to posttest (median 60.50, IQR 55.80‐64.30; *Z*=2.425; *P*=.02; Table 2 and Figure 3). To ensure robustness and account for the small sample size, a nonparametric bootstrap with 5000 replications was applied to calculate the 95% CI for the median reduction, which was 3.00 points (95% CI 1.00‐4.50). At the subscale level, the DIF subscale showed a marginal change toward significance (*Z*=1.826; *P*=.07), while no significant differences were observed for Difficulty Describing Feelings (*Z*=0.867; *P*=.39) or EOT (*Z*=0.200; *P*=.84). This suggests that the overall reduction in alexithymia was primarily driven by improvements in identifying feelings, while changes in other specific subdimensions did not reach statistical significance.

**Table 2.** Wilcoxon signed-rank test results of TAS-20 scores pre- and post-intervention.^f^

	Pretest median (IQR)	Posttest median (IQR)	Z	*P* value
Total				
TAS-20^a^	64.00 (59.50‐68.00)	60.50 (55.80‐64.30)	2.425	.02
Subscales				
DIF^b^	21.00 (18.00‐24.00)	17.50 (15.00‐21.50)	1.826	.07
DDF^c^	15.00 (13.80‐17.00)	14.00 (13.00‐16.30)	0.867	.39
EOT^d^	28.00 (26.80‐29.00)	27.50 (26.00‐29.00)	0.200	.84

a TAS-20: 20-item Toronto Alexithymia Scale.

b DIF: Difficulty Identifying Feelings.

c DDF: Difficulty Describing Feelings.

d EOT: Externally Oriented Thinking.

**Figure 4.**
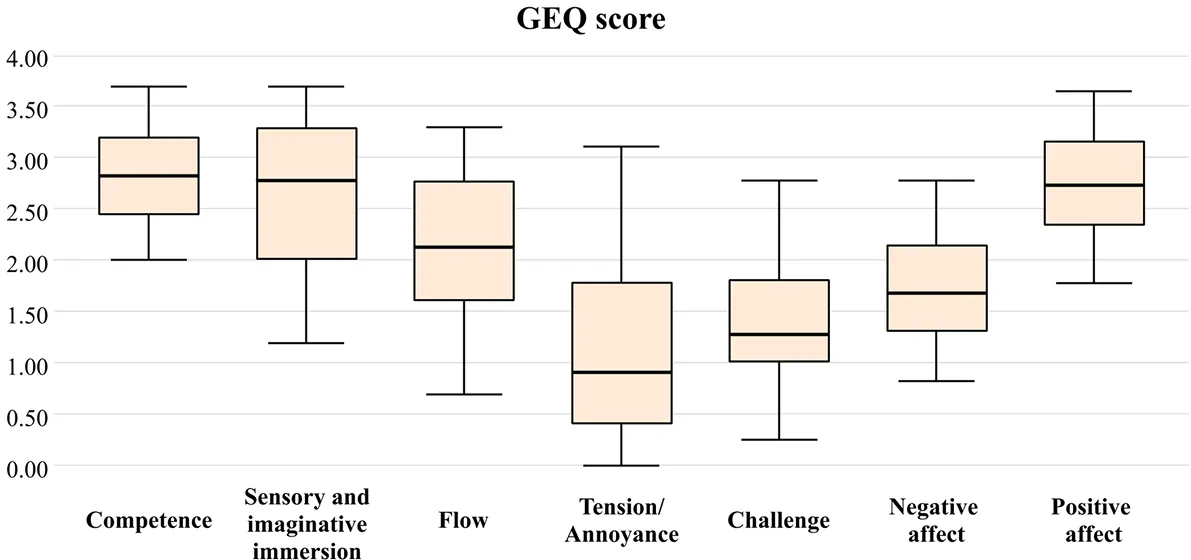
Boxplots of GEQ subscale scores. GEQ: Game Experience Questionnaire.

The quantitative results of the GEQ clearly demonstrate the participants’ gaming experience: competence (median 2.85, IQR 2.48‐3.20) was the highest-scoring dimension, indicating that participants felt efficient and capable while using the tool (Table 3 and Figure 4). This was followed by sensory and imaginative immersion (median 2.80, IQR 2.05‐3.28), showing that participants were well-immersed in the coloring game environment. Flow (median 2.15, IQR 1.63‐2.78) represented a moderate level, suggesting that participants’ immersion in the task flow was weaker than their immersion in the environment. Notably, tension or annoyance (median 0.90, IQR 0.40‐1.75) and challenge (median 1.30, IQR 1.00‐1.78) were the lowest, confirming that participants perceived the task as easy and stress-free. Finally, while negative affect (median 1.70, IQR 1.30‐2.15) remained low, positive affect (median 2.70, IQR 2.30‐3.13) was relatively high, indicating that participants generally considered the experience emotionally beneficial.

**Table 3.** Descriptive statistics of GEQ subscale scores.^a^

GEQ Subscales	Median (IQR)
Competence	2.85 (2.48‐3.20)
Sensory and imaginative immersion	2.80 (2.05‐3.28)
Flow	2.15 (1.63‐2.78)
Tension and annoyance	0.90 (0.40‐1.75)
Challenge	1.30 (1.00‐1.78)
Negative affect	1.70 (1.30‐2.15)
Positive affect	2.70 (2.30‐3.13)

a Scores range from 0 to 4.

#### Demographic Variables and Questionnaire Scores

To comprehensively investigate the potential impact of demographic variables and baseline characteristics on the game’s effectiveness and user experience, a series of nonparametric tests was performed for all collected factors. These included Mann-Whitney *U* tests for sex, education level, and academic major, as well as Kruskal-Wallis H tests for TAS-20 pretest severity level. The analysis revealed that only sex and TAS-20 pretest severity level exerted significant influences on specific dimensions of effectiveness, while no other demographic factors reached statistical significance. To maintain focus on meaningful findings, only significant outcomes are detailed below.

Significant sex differences were observed in two specific dimensions (Table 4). Regarding DIF, male participants (median 4.00, IQR 1.00‐8.00) exhibited a significantly more pronounced median reduction than females (median 0.00, IQR −3.00 to 3.00; *Z*=−2.137; *P*=.03). Conversely, regarding EOT, female participants (median 2.00, IQR −2.00 to 3.00) showed a greater median reduction compared with their male counterparts (median −1.00, IQR −2.00 to 0.00; *Z*=−1.998; *P*=.046).

**Table 4.** Mann-Whitney *U* test results of sex differences in TAS-20 score reductions.

	Male median (IQR)	Female median (IQR)	Z	*P* value
Total				
TAS-20^a^	3.00 (0.00‐6.00)	3.00 (0.00‐4.00)	−0.536	.59
Subscales				
DIF^b^	4.00 (1.00‐8.00)	0.00 (−3.00 to 3.00)	−2.137	.03
DDF^c^	0.00 (−2.50 to 2.00)	2.00 (−2.00 to 2.00)	−0.852	.39
EOT^d^	−1.00 (−2.00 to 0.00)	2.00 (−2.00 to 3.00)	−1.998	.046

a TAS-20: 20-item Toronto Alexithymia Scale.

b DIF: Difficulty Identifying Feelings.

c DDF: Difficulty Describing Feelings.

d EOT: Externally Oriented Thinking.

The pretest severity level significantly impacted the change in the EOT dimension (H=8.233; *P*=.02; Table 5). Specifically, participants in the “severe” group demonstrated a notable median reduction (median 3.00, IQR 2.00‐6.00), whereas the “definite” group followed a different pattern of variation (median −1.00, IQR −2.80 to 0.00), and the “borderline” group remained relatively stable (median 0.00, IQR −0.50 to 5.50). Further post hoc multiple comparisons were conducted for the EOT dimension to clarify pairwise differences. The “definite” group showed significantly different change scores relative to the “borderline” group (*P*=.04) and the “severe” group (*P*=.01), while no significant difference was detected between the “borderline” and “severe” groups (*P*=.40). Considering the conciseness of these pairwise findings, a separate post hoc comparison table was not included.

**Table 5.** Kruskal-Wallis H test results of pretest severity levels on TAS-20^a^ score reductions.

	Borderline median (IQR)	Definite median (IQR)	Severe median (IQR)	H	*P* value
Total					
TAS-20	2.00 (−1.00 to 3.00)	3.50 (−0.50 to 5.80)	3.00 (0.00 to 15.00)	1.353	.50
Subscales					
DIF^b^	0.00 (−4.00 to 2.50)	3.00 (0.30 to 7.00)	0.00 (−5.00 to 6.00)	3.521	.17
DDF^c^	0.00 (−1.50 to 1.00)	1.50 (−2.00 to 2.00)	3.00 (−2.00 to 5.00)	1.628	.44
EOT^d^	0.00 (−0.50 to 5.50)	−1.00 (−2.80 to 0.00)	3.00 (2.00 to 6.00)	8.233	.02

a TAS-20: 20-item Toronto Alexithymia Scale.

b DIF: Difficulty Identifying Feelings.

c DDF: Difficulty Describing Feelings.

d EOT: Externally Oriented Thinking.

### Qualitative Results

**Figure 5.**
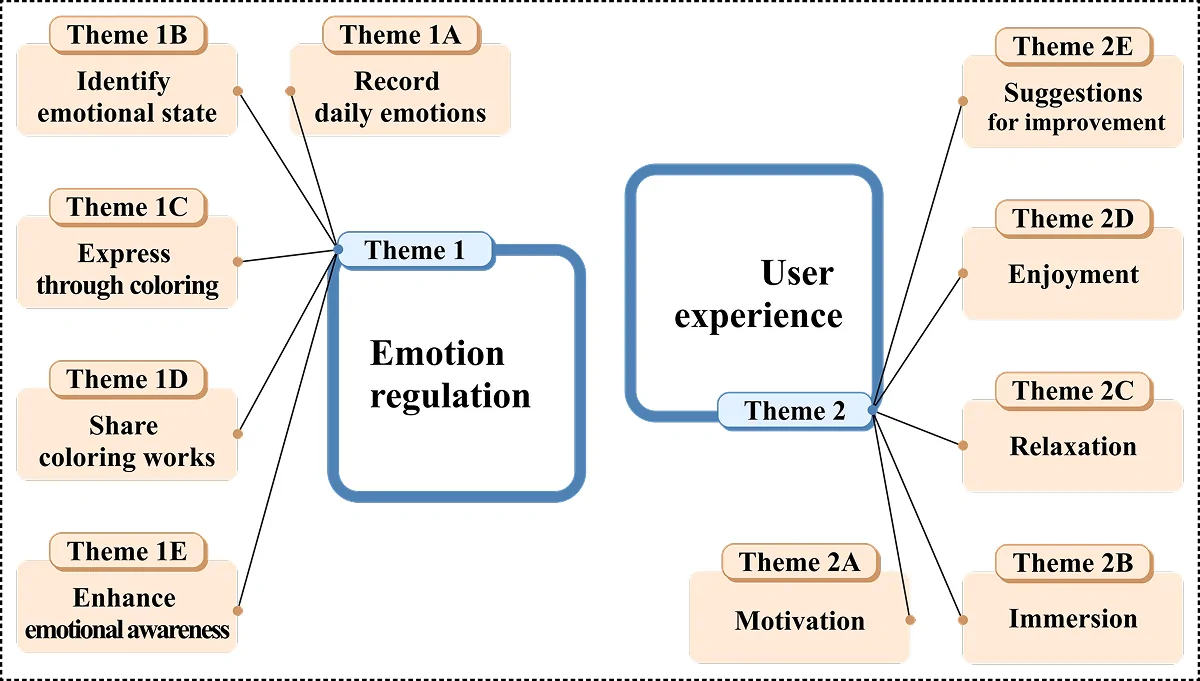
Thematic map of qualitative interview results.

Thematic analysis of the semistructured interviews yielded important insights into both the effectiveness and user experience of Coloring the Emoji for Chinese college students with alexithymia. Two major themes and 10 subthemes were identified, along with the covered codes (Figure 5 and Table 6).

**Table 6.** Themes and subthemes from qualitative analysis.

Theme and subthemes	Codes
Emotion regulation	
Record daily emotions	Immediate recording, emotionally salient moments, daily reflection, emotional release, reframing past experiences, sense of lightness
Identify emotional state	Color emotion association, self-reflection, positive feedback, expressing inner emotions, new opportunity for awareness
Express through coloring	Emotional release, catharsis, relief after expression, symbolic representation of anger, satisfaction through coloring, emotional recording
Share coloring works	Casual sharing, positive emotions for sharing, balancing openness and privacy, minimal sharing tendency, curiosity about others’ expressions
Enhance emotional awareness	Recognizing emotional patterns, forced expression leading to awareness, improved self-expression, greater ability to articulate feelings, linking emotions to daily events, accumulated reflection
User experience	
Motivation	Convenience, good results with less time and effort, achievement, sense of accomplishment, reflection, emotional linkage, history tracking
Immersion	Focus on simple tasks, soothing mood, commemorative value, aesthetic dissatisfaction, color & emotion mismatch, uniformity
Relaxation	Pressure release, emotional expression, mood improvement, relaxation through expression, venting feelings
Enjoyment	Improved mood, emotional expression, reflection on feelings, lightness, mood change through drawing
Suggestions for improvement	Avoid rigid templates, customizable color emotion mapping, free drawing options, custom emojis, meme-based creativity, balance between freedom and usability, statistical tracking features, emotional sticker wall, need for social interaction features, social engagement opportunities, brush usability

#### Theme 1: Emotion Regulation

##### Subtheme: Record Daily Emotions

Over three-quarters of participants (16/20) described the act of recording as a daily practice for revisiting and releasing emotions. They viewed it not simply as a tool for recording events, but also as a valuable way to reconstruct their immediate mental state and capture the emotionally salient moments. Importantly, many participants emphasized that this process also fostered reflection, allowing them to revisit past experiences from a different perspective.

*I felt some sentimentality. The problems that once troubled me no longer seem important; they have already passed, leaving me with a sense of lightness, as if I had crossed mountains with ease.*

[E7]

*Writing down the emotions of that moment, especially those tied to recent events that left a strong impression.*

[E10]

*If something particularly upsetting or striking happened during the day, I would record the emotions of that specific moment; if the day ended on a relatively ordinary note, I would reflect on the overall mood of the day.*

[E16]

##### Subtheme: Identify Emotional State

The majority of participants (15/20) found the method of representing emotions through the selection of colors and patterns both novel and engaging. This process transforms abstract emotions into concrete visual symbols, effectively reducing participants’ DIF. Through this mental mapping, previously vague bodily sensations become clearer and more distinct, providing participants with opportunities to process emotions and develop self-understanding, which directly enhances their emotion recognition abilities.

*I choose different colors based on my mood of the day, and then I select different expressions or patterns to draw.*

[E4]

*When I pick a color, I realize that I am reflecting on my current mental state... Even though what I create might not be very beautiful, the process itself gives me positive feedback.*

[E11]

*One good thing about it is that it provides some tools, which give me an opportunity I didn’t realize before. I can express my inner emotions through this kind of doodling.*

[E12]

##### Subtheme: Express Through Coloring

Many participants attempted to transform the emotions they identified into a form of psychological release through coloring. However, some participants (5/20) stated that they did not perceive this as a stress-relieving method and did not engage in deep reflection on their inner feelings. This tendency to prioritize objective facts over deep personal reflection suggests that the tool has a limited impact on shifting participants away from EOT. Furthermore, the fact that participants remained at the recording stage without achieving emotional release indicates that more support is needed to help participants effectively externalize and difficulty describing emotions.

*However, I might not have considered it as a decompression process, but rather as a record.*

[E13]

*For example, when I record my bad moods, I feel a slight sense of release after I finish... It’s like having someone to talk to, or like texting a good friend after we argued, which leaves me feeling a little better.*

[E15]

*For instance, when I’m angry, I select a punching or some other muscular expression, and then I pair it with a “strong punch” phrase. It actually feels kind of satisfying.*

[E16]

##### Subtheme: Share Coloring Works

Some participants (5/20) felt conflicted when sharing their work, worrying about expressing themselves inappropriately or being misunderstood. Due to difficulty in navigating the boundaries of sharing, most chose to remain silent or reduce interaction. This social withdrawal behavior indicates that participants are still avoiding deep emotional interaction, and therefore, their EOT scores did not improve significantly.

*Sometimes, I get curious about what others want to express, especially when I see something intriguing, but I’m not sure what they are trying to communicate.*

[E7]

*I feel like, when I draw, I might as well share it. There’s no harm in sharing, so I just do it. Actually, I haven’t really thought too much about it.*

[E13]

*I feel that positive emotions can be shared with others, but I think the difficult part about sharing is knowing the right balance. It’s hard to gauge the appropriate level, so I tend to share less, keeping it to a minimum.*

[E13]

##### Subtheme: Enhance Emotional Awareness

A significant proportion of participants (8/20) indicated that consistent drawing practice helped improve their emotional awareness and expression. Over time, many participants developed a habit of recording their emotions, with some even stating that the process had become an essential part of their daily lives.

*Well, if you don’t draw, you might not realize what’s going on. But once you start drawing, you realize, wow, it’s like I’ve been drawing the same thing every day.*

[E3]

*It’s like there’s something that forces me to express what I’m feeling now. It’s this feeling where you don’t want to express it, but you must. Since I’m doing this, I must express it.*

[E6]

*I’m naturally someone who doesn’t talk much, and I might have some sort of expressive difficulty. But recently, they told me that I’ve been talking more, expressing myself more.*

[E8]

*I can relate the expression of the day to what happened to me that day, and the more I accumulate, the more interesting it becomes.*

[E16]

### Theme 2: User Experience

#### Subtheme: Motivation

Participants’ motivation primarily stems from a high sense of competence and low psychological barriers. Because the tool is intuitive and easy to use, most participants (11/20) achieve satisfying visual results with minimal time and effort. The low challenge level eliminates the pressure of daily recording, making it easier for participants to maintain the habit and focus on their emotional expression. By accumulating these “microachievements,” the daily ritual shifts from a perceived task into a satisfying and effortless behavioral habit.

*I found a way to draw that is convenient and produces good results with less time and effort… it allows for color separation, feels fast to do, and doesn’t look bad.*

[E10]

*When it goes well, playing this feels like a small achievement... even if it doesn’t turn out well, completing it still gives a sense of accomplishment.*

[E10]

*Recording every day and looking back at the history is very interesting. Linking the expressions to what happened that day and seeing them accumulate gives me a real sense of achievement.*

[E16]

#### Subtheme: Immersion

For some participants (6/20), immersion stemmed primarily from the “quiet atmosphere” created by the game. While a simple visual environment can help students focus on their emotions, the coloring task itself was too mechanical and lacked variation, limiting participants from entering a deep flow state. This explains why immersion scores were high while flow scores were low: the game environment successfully kept participants “staying,” but the single interactive task was not enough to make them “forget about time.”

*I think the ugliness definitely affects me, but the ugliness is just a result. The issue is that this activity, combining the mosaic-style blocks with the predefined emojis, is quite uniform. Most of the outcomes are very similar.*

[E5]

*I feel that simply focusing on a simple task, doing it calmly, naturally, helps to soothe my mood.*

[E10]

*I have a rather strange feeling when recording negative emotions; after finishing, I feel a sense of self-pity, which is quite an odd sensation. However, when recording happiness, it feels more like it has commemorative value, as if I’m putting a stamp on today’s calendar.*

[E12]

#### Subtheme: Relaxation

Most participants (14/20) experienced significant relaxation. The game is simple and easy to master, allowing participants to observe real-life stresses through drawing without any psychological burden. For example, when reviewing their work, participants could perceive recent accumulated mental fatigue from changes in color preferences. Simultaneously, the coloring activity provided an effective outlet, allowing participants to externalize and release discomfort. This easy-to-learn game experience, combined with its emotional externalization function, effectively reduced the overall psychological burden on participants.

*When I look back at my paintings recently, I realize that I was probably under a lot of pressure and had a lot to do last week, so I painted some works with a cooler tone.*

[E4]

*I think there has been an improvement. Sometimes when I’m in a bad mood or feeling a little uncomfortable, drawing helps me release those feelings. It makes me feel much more relaxed when I can vent and express myself through this coloring game.*

[E7]

#### Subtheme: Enjoyment

Many participants (8/20) experienced a significant positive emotional boost after completing their artwork. This sense of pleasure stemmed from the fact that painting provided participants with an opportunity for reflection and catharsis. By transforming discomfort into visual expression, participants felt a reduction in psychological burden and a greater sense of ease after completion. This inner peace also had a positive impact on daily life; for example, some introverted participants found themselves becoming more proactive in real-life social interactions, more willing to communicate and share their feelings with others.

*I’m usually not a very talkative person, but when I was talking to my girlfriend recently, she said that I seemed to be talking more and expressing myself more.*

[E8]

*I think it does improve my mood. Sometimes when I’m in a bad mood or feel a little uncomfortable, drawing helps me release those feelings. It makes me feel much lighter by expressing myself through drawing.*

[E9]

*I don’t think my good or bad mood changes because of the process of drawing, but drawing reminds me to reflect on my feelings, and I feel much lighter after finishing.*

[E10]

#### Subtheme: Suggestions for Improvement

Regarding suggestions for improvement, participants’ feedback mainly focused on the following 3 aspects. First, enhancing creative freedom and aesthetic experience (5/20). Participants hoped to break free from the limitations of fixed templates, be able to customize the correspondence between colors and emotions, and increase the selection of more graphics; they also suggested improving usability issues, such as brush feel, and maintaining appropriate guidance while increasing freedom to balance operational difficulty and creative enjoyment. Second, adding a personalized data feedback system (3/20). Participants desired to see their progress more directly, such as introducing statistical charts or line graphs to record daily login frequency and emotional change trends, using data-driven feedback to enhance self-control and long-term sense of accomplishment. Finally, building a deep social interaction community (4/20). Participants were not satisfied with simple work display and hoped to build a real community with features such as leaving messages and comments, and proposed highly interactive features, such as creating custom emoticons or using popular “memes” for secondary creation, to enhance the platform’s long-term appeal.

*I don’t want to use templates. I like to define my own colors for emotions. ...I’d also like the subsequent images to follow my personal color palette. I hope the interface allows for free drawing and simplifies the background template.*

[E1]

*The intuitive connection between color and emotion doesn’t match. For example, the color I like corresponds to an emotion I don’t like. The images aren’t aesthetically pleasing and are too abstract.*

[E1]

*I really hope the platform develops into a real social community, not just a platform for display. Right now, it lacks interactive features like messages and comments, so it doesn’t feel like a true social platform.*

[E1]

*It doesn’t seem too difficult, but it’s more about consistency. The frame feels a bit limited, and there are a few shapes to choose from.*

[E2]

*I’d like to see something like a statistical graph, such as a line chart that shows how many times I logged in today, tomorrow, and the day after.*

[E5]

*I feel like the pixel brush isn’t very easy to use.*

[E7]

*One really appealing way is creating custom emoji packs, like making fun emojis that could include text, or using popular meme images. You could take these memes and add your own creative touches.*

[E8]

*There’s a balance here. If the freedom is great, it becomes too complicated to draw, which could drive some people away. We need a middle ground where there’s a certain degree of freedom.*

[E10]

*But if you just give me a blank sheet, I wouldn’t know what to draw. I think guidance is necessary, but I’m not sure how much guidance or restrictions should be in place.*

[E12]

### Integration of Quantitative and Qualitative Results

Using a subgroup-comparative joint display, this study integrated quantitative and qualitative findings (Table 7). Quantitative analyses revealed significant differences in TAS-20 changes across subgroups; males showed a greater reduction in the DIF subscale, while females improved more in the EOT subscale. Both the borderline and severe alexithymia groups exhibited more pronounced reductions in EOT compared with the definite alexithymia group. Qualitative themes explained these disparities; sex differences reflected distinct usage patterns, with males favoring expression-focused engagement and females preferring reflection-focused interaction; improvements in the borderline group were linked to high perceived control and positive perceptions of the tool as a lightweight emotional aid, while benefits in the severe group were associated with stronger therapeutic reliance and goal-oriented use.

**Table 7.** Joint display: group differences and corresponding interpretations.

Subgroup comparison	Quantitative outcome	Qualitative theme	Integrated interpretation
Males vs females	Males showed significantly greater reduction in DIF^a^ (*P*=.03); females showed significantly greater reduction in EOT^b^ (*P*=.046).	Males: expression-focused use (daily emotional recording). Females: reflection-focused use (reviewing coloring works).	Sex differences align with interaction styles: expression vs reflection drives divergent benefits.
Borderline vs definite alexithymia	Borderline group showed significantly greater reduction in EOT (*P*=.04).	Borderline: high perceived control, lightweight tool use. Definite: low perceived control, negative usability experiences.	Higher control vs lack of control explains greater improvement in the Borderline group.
Severe vs definite alexithymia	Severe group showed significantly greater reduction in EOT (*P*=.01).	Severe: high therapeutic reliance, goal-oriented use. Definite: low therapeutic reliance, weaker user experience.	Stronger reliance vs low engagement drives greater benefits in the Severe group.

a DIF: Difficulty Identifying Feelings.

b EOT: Externally Oriented Thinking.

## Discussion

### Principal Results

This prospective, exploratory convergent parallel mixed methods longitudinal study was conducted to address 3 predefined objectives: evaluate the effectiveness of Coloring the Emoji in reducing alexithymia symptoms, examine its user experience as a daily emotion regulation tool, and provide empirical support for coloring-based digital art therapy in alexithymia intervention. Overall, the 15-day intervention significantly reduced overall alexithymia, with the most prominent improvement observed in the ability to identify feelings. Through a subgroup-comparative joint display, this study further integrated quantitative and qualitative findings to interpret heterogeneous intervention outcomes. Subgroup analysis identified divergent TAS-20 improvements by sex and baseline alexithymia severity, while qualitative themes contextualized these differences through distinct usage patterns, personal perceptions, and intervention motivations across groups. User experience was consistently favorable, characterized by high competence, strong immersion, low psychological pressure, and broad acceptability across demographic subgroups. Qualitative findings further illuminated the underlying mechanisms of change, including visual symbolization of emotions, daily reflective practice, and low-pressure engagement. Collectively, these results achieved all 3 study objectives and provide preliminary empirical evidence supporting the feasibility, acceptability, and potential effectiveness of digital coloring games as a low-threshold emotion regulation approach for Chinese college students with alexithymia.

### Effectiveness

The intervention significantly reduced overall alexithymia, with the most notable changes observed in the ability to identify feelings. This pattern aligns with theoretical and empirical work indicating that visual, nonverbal expression facilitates the concretization of vague affective sensations, a core challenge in alexithymia [24,25,53-55]. Although measurable progress was seen across all TAS-20 dimensions, changes in emotional description and externally oriented thinking did not reach statistical significance, indicating that rigid cognitive tendencies related to alexithymia require longer or more targeted intervention to achieve long-term improvement [31,56]. Consistent with these quantitative trends, qualitative accounts further validated such intervention effects. Most participants acknowledged that translating inner feelings into colors and visual symbols helped organize ambiguous emotions and enhance emotional clarity, which directly explained the significant improvement in identifying feelings. At the same time, limited depth of daily usage and cultural restraint regarding emotional disclosure restricted more fundamental shifts in cognitive styles and emotional expression habits [57,58]. Collectively, these outcomes indicate Coloring the Emoji functions effectively as an entry-level intervention for emotional awareness rather than a comprehensive treatment for all alexithymic features.

Subgroup differences in therapeutic responses were further clarified through integrated quantitative and qualitative analysis. Sex and baseline alexithymia severity acted as key moderating factors, showing differentiated improvement pathways across participants. Males obtained greater reductions in difficulty identifying feelings, which corresponded to their preference for intuitive, expression-driven visual engagement, while females benefited more in reducing externally oriented thinking through long-term reflective practice [59]. In terms of clinical severity, participants with borderline and severe alexithymia showed more favorable improvements in externally oriented thinking compared with those with definite alexithymia. Such disparities can be explained by distinct psychological needs and usage motivations: borderline participants regarded the tool as a lightweight and controllable daily aid, whereas individuals with severe alexithymia engaged with the intervention in a more goal-oriented manner to alleviate emotional avoidance [60-63]. These integrated findings provide a nuanced understanding of how user characteristics shape intervention outcomes, supporting the personalized value of low-pressure creative interventions for different vulnerable groups.

### User Experience

User experience was strongly positive, marked by high competence, immersion, and positive affect, coupled with low tension and challenge. This profile reflects a key design strength: minimizing performance pressure while supporting consistent engagement [47]. The absence of significant demographic differences in user experience indicates broad accessibility across sex, education, and academic background. Moderate flow scores suggest that repetitive, template-based tasks limited deep absorption, highlighting opportunities for design refinement. Qualitative feedback further clarified user experiences and improvement needs. Participants attributed high competence to effortless “microachievements” in daily completion. Common suggestions included greater creative autonomy, personalized data feedback, and privacy-preserving social interaction. These insights provide empirically grounded directions for enhancing long-term engagement while respecting cultural preferences for cautious emotional disclosure.

### Comparison With Previous Work

First, this study demonstrates that a digital coloring medium can effectively overcome the practical barriers inherent in traditional art therapy. While previous research has established the therapeutic value of conventional art therapy in fostering emotional awareness [53,54], its broader implementation is often hindered by spatial constraints, high costs, and participants’ resistance to “clinical” settings [31,55]. *Coloring the Emoji* addresses these challenges through a “de-medicalized” design that preserves the core expressive functions of art therapy while ensuring high engagement through its low-barrier entry. These findings align with digital mental health frameworks and reinforce the feasibility of adapting art therapy into everyday digital tools to expand the reach of population-level mental health interventions [36-38].

Furthermore, our findings refine the understanding of how digital interventions impact the distinct dimensions of alexithymia. The improvement in DIF aligns with previous evidence suggesting that visual-to-emotion mapping facilitates the concretization of abstract emotion [60,61]. However, the relative stability of EOT scores echoes a common challenge in digital health: the difficulty of modifying deep-seated cognitive styles through short-term, low-intensity interventions [62,63]. Beyond general intervention effects, the integrated subgroup analysis of this study further reveals heterogeneous responses across sex and baseline alexithymia severity, indicating that user characteristics shape intervention pathways and therapeutic gains [64]. Such differentiated outcomes have often been overlooked in previous digital art therapy studies, which tend to report overall group effects rather than individualized patterns of improvement [65,66].

Finally, this work contributes a nuanced cross-cultural perspective to a field where interventions are predominantly evaluated within Western contexts [57,58]. While many Western frameworks emphasize active emotional externalization and social sharing, the “ambivalent sharing” observed among Chinese college students, characterized by a desire for connection tempered by a cautious approach to emotional disclosure, highlights variations in user preferences across different cultural landscapes. Rather than dismissing the value of social design, these findings suggest that future global interventions should incorporate more flexible, private, and semianonymous interaction structures to better align with diverse cultural expectations regarding emotional safety [67,68].

### Limitations

This study has several limitations. First, as an exploratory intervention, the current study period primarily captured transient psychological shifts and user engagement patterns. While the initial results are promising, the long-term stability of the therapeutic impact requires further validation by extending the follow-up period. Second, although independent double coding was adopted to improve the credibility of qualitative analysis, formal interrater reliability testing was not performed, which may introduce subjective bias to thematic interpretation. Third, the sample consisted of a relatively homogeneous group of college students within a specific cultural context. Future research should expand the diversity of the cohort to include a broader range of age groups, professional backgrounds, and cultural environments to fully verify the generalizability and adaptability of this digital art therapy tool across diverse populations.

### Conclusions

This study provides preliminary empirical evidence that Coloring the Emoji, a coloring-based serious game, is a feasible and culturally appropriate digital art therapy for Chinese college students with alexithymia. Its core innovation lies in the targeted validation of a localized, low-threshold coloring-centered art therapy tool tailored for Chinese college populations. Differing from Western-centric interventions that prioritize open emotional expression and social sharing, this study delivers culture-sensitive evidence adapted to the reserved emotional norms of Chinese college students. It advances the field by enriching empirical research on nonverbal, creative digital interventions for alexithymia and supporting the broader application of light-intensity digital art therapy in youth mental health. In practical terms, this demedicalized, easy-access tool offers actionable, real-world value for campus mental health promotion, serving as a scalable daily emotion regulation supplement to reduce barriers to traditional mental health services and meet the emotional needs of contemporary college students.

## Supplementary material

10.2196/83540 Multimedia Appendix 1 Instruments of the experiment.

10.2196/83540 Multimedia Appendix 2 Interview guide.

## Abbreviations

DIF: Difficulty Identifying Emotions
EOT: Externally Oriented Thinking
GEQ: Game Experience Questionnaire
TAS-20: 20-item Toronto Alexithymia Scale
