# Supplementary material for: Coloring-Based Serious Game in Chinese College Students With Alexithymia: Prospective Longitudinal Exploratory Mixed Methods Study
Source: JMIR Serious Games. 2026 Aug 19;14:e83540. doi: 10.2196/83540 (PMC13488921; doi:10.2196/83540)
Supplement: Supplementary file 1 [file games-v14-e83540-s001.docx]

## Multimedia Appendix 1: Instruments of the experiment.

### Introduction video

The video introduces alexithymia through the story of Lily, a young woman who struggles to express her emotions, highlighting a common challenge faced by many Chinese college students. *Coloring the Emoji* supports emotion recognition and expression through intuitive coloring tasks. Featuring minimalist pixel art design, the game combines color selection, pattern selection, and free coloring to create a non-verbal, low-pressure environment, enhancing ease of use and reducing emotional defensiveness.

Figure 1. Background section from the Introduction video

### User Manual.

*Coloring the Emoji* is a coloring-based serious game designed to support users with alexithymia in emotion regulation. The game offers an intuitive, non-verbal platform where users can process their emotions through color selection, pattern selection, and free coloring. The overall process is shown in the user manual (Figure 2).

Figure 2. User Manual.

### Interface Navigation

*Coloring the Emoji* relies on the WeChat mini-program platform, and users can access it through it. Once signed in, users can experience the core functions of the application in the following sequence:

1. **Color Selection** - Use the color palette to select preferred colors associated with the user's emotions, with up to three color options.
2. **Pattern Selection** - Select one of a set of predefined emoji templates to serve as the basis for subsequent coloring activities.
3. **Freely Coloring** - Apply the selected color to the selected template to generate pixel-style coloring works.
4. **Save Coloring Works** – Store the completed coloring works within the application using the save function.
5. **Coloring Works History** – Access and manage previously saved coloring works through the History Page.
