# Supplementary material for: Coloring-Based Serious Game in Chinese College Students With Alexithymia: Prospective Longitudinal Exploratory Mixed Methods Study
Source: JMIR Serious Games. 2026 Aug 19;14:e83540. doi: 10.2196/83540 (PMC13488921; doi:10.2196/83540)
Supplement: Supplementary file 2 [file games-v14-e83540-s002.docx]

## Multimedia Appendix 2: Interview guide.

**Interviewer instructions:** To collect participants’ feedback on their experiences and perceptions of *Coloring the Emoji* during the experiment：

1. Use open-ended questions to encourage detailed responses.
2. Focus on participants’ emotional experiences, regulation strategies, and perceptions of the game, prioritizing depth over breadth.
3. Allow participants to skip or pause questions at any time to ensure comfort.
4. Conduct interviews via Zoom on day 1 (December 8, 2024), day 8 (December 16, 2024), and day 15 (December 23, 2024) of the intervention.

### Interview Guide – Session 1 (≤10min)

**Introduction**

Thank you for joining today’s interview. This session will focus on your previous experiences with emotion regulation and related tools. Please feel free to share openly, and remember you may skip or pause at any time.

**Self-Emotion Regulation Experience**

1. Have you used similar emotion regulation tools or counseling services before?
2. If so, could you share your experience? For example:

- Self-emotion regulation: Journaling, meditation, or deep breathing.
- Emotional timers or mood apps: Recording mood changes or using emotion management apps.
- Psychological counseling or psychotherapy: Professional counseling or therapy.

**Overall Reflection**

3. How was your overall experience?
4. What methods did you find effective and ineffective?
5. Can you describe 1-2 memorable experiences?

**Conclusion**

Thank you for sharing your experiences today. Your input is very valuable for our study. If you have any additional thoughts later, please feel free to share them with us.

### Interview Guide – Session 2 (10min-15min)

**Introduction**

Thank you for joining us for Part 2 of today's interview. In this part, we'd like to learn more about your experience with *Coloring the Emoji*, your interactions with your coloring works and social features, and any changes you may have noticed in your emotion regulation strategies. Please feel free to share your thoughts and remember that you can skip or pause at any time.

**Daily Online Experience**

1. Were you able to consistently access the game daily? Did you encounter any difficulties?
2. On average, how many coloring works do you complete in a day?

**Interaction With Coloring Works**

3. How do you feel about your coloring works?
4. Did you frequently review your coloring works?
5. Did you frequently browse the coloring works of others in the community? How did you feel about this interaction?

**Changes in Emotion Regulation**

6. How is your overall gaming experience now?
7. Do you think your emotion regulation strategies have changed so far?

**Conclusion**

Thank you for sharing your experiences today. Your input is very valuable to us. If you have any additional thoughts later, please feel free to share them with us.

### Interview Guide – Session 3 (20min-30min)

**Introduction**

Thank you for participating in our final interview today. This interview will focus on your overall experience with *Coloring the Emoji*, the impact of coloring on your emotional regulation, your interactions with coloring pieces and social features, and factors that influence your engagement. Finally, we will ask for your suggestions for future improvements to the game. Please feel free to share your feedback and remember that you can skip or pause at any time.

**Overall Experience**

1. Were you able to consistently access the game daily? If not, what were the main reasons?
2. How was your overall gaming experience?
3. Did the game meet your expectations?

**Impact on Emotion Regulation**

4. Did the frequency and duration of play affect your emotion regulation?
5. Did your emotional state improve throughout your participation?
6. Did you feel more able to manage your emotions after completing this process?
7. In what ways did the game better support your emotion regulation?

**Coloring Works and Social Features**

8. Did you review your coloring works frequently? If so, did you gain new insights during this review process?
9. Did you find that completing and reviewing your coloring works helps improve your emotion? If so, what were those insights?
10. Did social interaction enhance your ability to regulate emotions? Did it motivate you to stay online?

**Influencing Factors**

11. What factors helped you complete your overall participation over the 15 days? Did external support or incentives play a role?

**Future Improvements**

12. If you continue to use this game, please list the top 3 features you would like to see improved or added.

**Conclusion**

Thank you very much for your time and thoughtful feedback throughout this study. Your participation was crucial to our understanding of how this game supports emotion regulation. We hope that our game will continue to support your emotional management. We sincerely appreciate your contribution and wish you all the best in your future emotional well-being journey.
